# Function of *PHEX* mutations p.Glu145* and p.Trp749Arg in families with X-linked hypophosphatemic rickets by the negative regulation mechanism on *FGF23* promoter transcription

**DOI:** 10.1038/s41419-022-04969-5

**Published:** 2022-06-02

**Authors:** Yu-mian Gan, Yan-ping Zhang, Dan-dan Ruan, Jian-bin Huang, Yao-bin Zhu, Xin-fu Lin, Xiao-ping Xiao, Qiong Cheng, Zhen-bo Geng, Li-sheng Liao, Fa-qiang Tang, Jie-wei Luo

**Affiliations:** 1 Shengli Clinical Medical College of Fujian Medical University, Fuzhou, 350001 China; 2 Fujian provincial hospital, Fuzhou, 350001 China; 3 Department of Traditional Chinese Medicine, the First Affiliated Hospital, Fujian Medical University, Fuzhou, 350001 China; 4 Department of Obstetrics and Gynecology, Peking Union Medical College Hospital, Chinese Academy of Medical Sciences, Beijing, 100730 China

**Keywords:** Kidney diseases, Endocrinology

## Abstract

X-linked hypophosphatemic rickets (XLH) is characterized by increased circulating fibroblast growth factor 23 (FGF23) concentration caused by *PHEX* (NM_000444.5) mutations. Renal tubular resorption of phosphate is impaired, resulting in rickets and impaired bone mineralization. By phenotypic-genetic linkage analysis, two *PHEX* pathogenic mutations were found in two XLH families: c.433 G > T, p.Glu145* in exon 4 and c.2245 T > C, p.Trp749Arg in exon 22. Immunofluorescence showed that the localization of p.Glu145* and p.Trp749Arg mutant and secretory PHEX (secPHEX) changed, with decreased expression. In a HEK293T cell model co-transfected with *PHEX*, sec*PHEX*, and *FGF23*, wild-type PHEX, secPHEX, and FGF23 proteins were distributed in the cell membrane or endoplasmic reticulum, while the mutant was located in the nuclear membrane and cytoplasm. qPCR of p.Glu145* revealed decreased *PHEX* and sec*PHEX* mRNA expression in cells, with no difference in mRNA expression of p.Trp749Arg. Both mutations decreased intracellular PHEX endopeptidase activity. Western blot analysis showed decrease in mutant and secPHEX protein expression and no FGF23 protein expression in single-transfected PHEX and secPHEX cells. In cells co-transfected with FGF23, *PHEX* and sec*PHEX* mutation promoted FGF23 expression. Dual-luciferase reporter gene was used to detect the effect of *PHEX* on *FGF23* promoter. The dual-luciferase reporter gene showed that after PHEX overexpression, the activity of mutant firefly luciferase was significantly higher than that of wild type. The regulatory mechanism between PHEX and FGF23 is still unclear, but we found that PHEX is a direct transcriptional inhibitor of FGF23 and affects the expression of FGF23. This study verified the pathogenicity of the two variants and revealed the possible regulatory mechanism between PHEX and FGF23.

## Introduction

X-linked dominant hypophosphatemic rickets (XLH, MIM307800) is the most common type of human hereditary rickets [1, 2]. XLH is characterized by increased urinary phosphorus excretion and decreased renal tubular phosphorus reabsorption rate, resulting in hypophosphatemia of renal phosphate consumption and abnormal vitamin D metabolism, thereby causing bone mineralization disorders [3–5]. In 1995, the XLH Consortium identified a phosphate-regulating gene with homologies to endopeptidases on the X-chromosome (*PHEX*, NM_000444.5), as the pathogenic gene of XLH [6]. The gene is located on chromosome X (Xp22.11), contains 22 exons, and has a full-length size of 2,861 bp. The protein encoded 749 residues (NP_000435.3). Its structure is composed of a short-chain intracellular domain, a transmembrane domain, and a large extracellular domain containing a zinc-binding region and conserved cysteine residues [7–9]. A unique hydrophobic peptide (SA domain) at the N-terminal not only guides protein transport through the rough endoplasmic reticulum as a signal peptide, but also anchors the protein to the plasma membrane of the cell as a transmembrane domain [10]. Secretory *PHEX* (sec*PHEX*) designed in our experiment refers to the use of gene-directed mutagenesis to convert the SA domain of *PHEX* into a signal peptide with cleavage activity. It is mainly used to explore the substrate of the PHEX enzyme [11]. Although PHEX expresses a protease, it mainly affects the expression of fibroblast factor 23 (FGF23), rather than promoting the degradation of FGF23 [12]. In mice and cell culture, although PHEX mRNA and/or proteins have been detected in tissues, such as the lung, brain, muscle, gonad, skin, and parathyroid gland [13, 14], PHEX has also been found in the bones and teeth. The expression of PHEX in bone is limited to the osteoblast lineage, osteoblasts, and osteocytes, but not in renal epithelial cells [14, 15]. Studies have shown that the phenotypes of PHEX knockout mice and Hyp mice overlap, with hypophosphatemia, high FGF23, and osteomalacia phenotypes [15].

FGF23, which is mainly produced by bone cells and osteoblasts, is a secretory protein containing 249 residues, and has a signal peptide comprising 24 residues, which is one of the core hormones of calcium and phosphate regulation [16]. The excess and deficiency of FGF23 lead to hypophosphatemia and hyperphosphatemia, respectively [17]. FGF23 mainly plays a role as a phosphate-regulating factor in the kidney, parathyroid gland, and other tissues, by downregulating the expression of Na/Pi in renal proximal convoluted tubules and 1 α-hydroxylase expression, thereby reducing phosphate resorption and affecting intestinal phosphate transport [18, 19]. Pathogenic variants in *PHEX* led to an increase in FGF23 concentration [20–23]. In addition, Burosumab, a human cloned antibody against FGF23, has also been approved for effective use in patients with XLH [24, 25]. In the initial study, FGF23 was thought to be directly regulated by endopeptidase encoded by *PHEX*, but it has been confirmed that FGF23 is not the direct substrate of the PHEX protein [26]. Addison et al. [27] proposed the concept of Proteaseresistant cell-serine-aspartate-rich motif (ASARM), believing that osteoblasts of HYP mice can secrete an unknown factor, referred to as minhibin, to inhibit the mineralization of the extracellular matrix. Minhibin is a theoretical substrate for PHEX. The ASARM contained in MEPE is a candidate for minhibin. Bone mineralization is regulated by various factors, while ASARM is a protein degradation product. PHEX plays the role of ASARM, where its inactivation leads to the accumulation of ASARM [27–29]. However, the regulatory mechanism between *PHEX* and *FGF23* is currently unclear.

Secretory PHEX (secPHEX) refers to the use of gene-directed mutagenesis to convert the SA domain of PHEX into a signal peptide with cleavage activity. It is mainly used to explore the substrate of the PHEX enzyme, and its main advantage is that it is easy to purify from the used medium without the need for detergent. It has been reported that there are differences in the properties of enzymes encoded by secPHEX and wild-type PHEX [11]. In this study, differences in function between the secPHEX gene produced by the SA domain mutation, and the enzyme expressed by the wild-type secPHEX gene were determined based on the pathogenic mutation of PHEX. We constructed different types of PHEX overexpression vectors, FGF23 expression vectors and dual-luciferase expression vectors to transfect HEK293T cells. The aim is to explore the cellular functions of p.Glu145* and p.Trp749Arg and the correlation between PHEX and FGF23.

## Results

### Case presentation

**Fig. 1:**
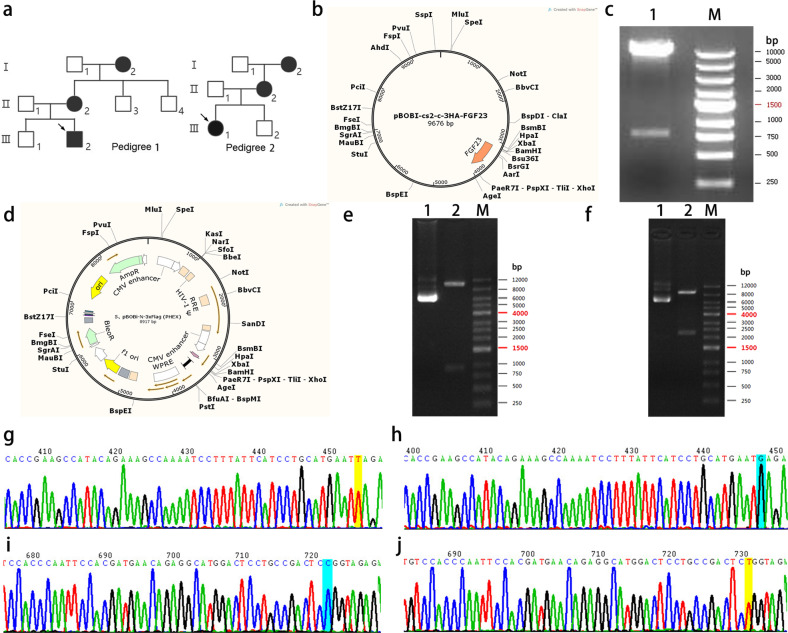
Pedigree map and plasmid construction of p.145Glu* and p.Trp749Arg. **a** X-linked dominant hypophosphatemic rickets (XLH) pedigree diagram. **b** pBOBI-cs2-c-3HA-*FGF23* plasmid vector schematic diagram (version of SnapGene 5.2; URL: https://www.snapgene.com/). **c** *FGF23* cloned plasmid identified by double enzyme digestion. Lane 1 is plasmid digested with BamHI/XhoI; Lane 2 is DNA marker. **d** pBoBi-N-3*Flag-*PHEX* plasmid vector schematic diagram (version of SnapGene 5.2; URL: https://www.snapgene.com/). **e** *PHEX* cloned plasmid identified by double enzyme digestion. Lane 1 is plasmid; Lane 2 is plasmid digested with XbaI-BsrGI; and Lane M is DNA marker. **f** sec*PHEX* cloned plasmid identified by double enzyme digestion. Lane 1 is plasmid, Lane 2 is plasmid digested with XbaI-AgeI, Lane M is DNA marker. **g**–**j** *PHEX* cloned gene sequencing map; **g** c.433 T (p.145*), 145 mut type; **h** c.433 G (p.145Glu*), 145 WT type; **i** c.2245 C(p.Trp749Arg), 749 mut type; and **j** c.2245 T (p.Trp749Arg), 749WT type.

In the author’s previous clinical practice, one novel mutation was identified in the genetic analysis of two Han X-linked hypophosphatemic rickets (XLH) families, where the probands clinically manifested gait instability, square skull, costal margin valgus, tooth loss, bracelet, and late tooth emergence. Serum phosphorus was significantly decreased (0.7, 0.69 mmol/L), and 24 h urine phosphorus was significantly increased (68.4, 58.0 mmol/day, respectively). X-rays of the limbs and chest showed signs of partial rickets: the epiphysis was found to be sunken and cupped (Mouth-like), there were brush-like changes, thin bone cortex, osteopenia, and bending deformity of the tibia. In the family survey, it was learned that there were two or three patients in each family, who were short, or had been diagnosed with rickets (Fig. 1a) in infancy or childhood. A heterozygous nonsense variant c.433 G > T (p.Glu145 *) in exon 4 of *PHEX* (NM_000444.5) was identified in pedigree 1, and a heterozygous missense variant c.2245 T > C (p.Trp749Arg) in exon 22 of *PHEX* was identified in pedigree 2 [22]. In line investigations and genetic screening were consistent with family genetic characteristics. According to the clinical phenotypic-genetic association analysis of the family, these two sites were considered to be responsible variants [22].

### Double enzyme digestion and sequencing verification of plasmid

After cloning the *PHEX*-WT, *PHEX*-mut, sec*PHEX*-WT, sec*PHEX*-mut, and *FGF23* genes, the genes were packaged into a lentiviral vector, transformed into *Escherichia coli* cells, and subjected to double enzyme digestion (Fig. 1c, e, f), and sequenced (Fig. 1g–j). The *PHEX*-WT, *PHEX*-mut, sec*PHEX*-WT, and sec*PHEX*-mut genes were successfully cloned and compared with known sequences in the National Center for Biotechnology Information (NCBI), and the results were consistent. Thus, these plasmids can be used in subsequent experiments.

### Immunofluorescence localization of PHEX, secPHEX, and FGF23 proteins in cells

**Fig. 2:**
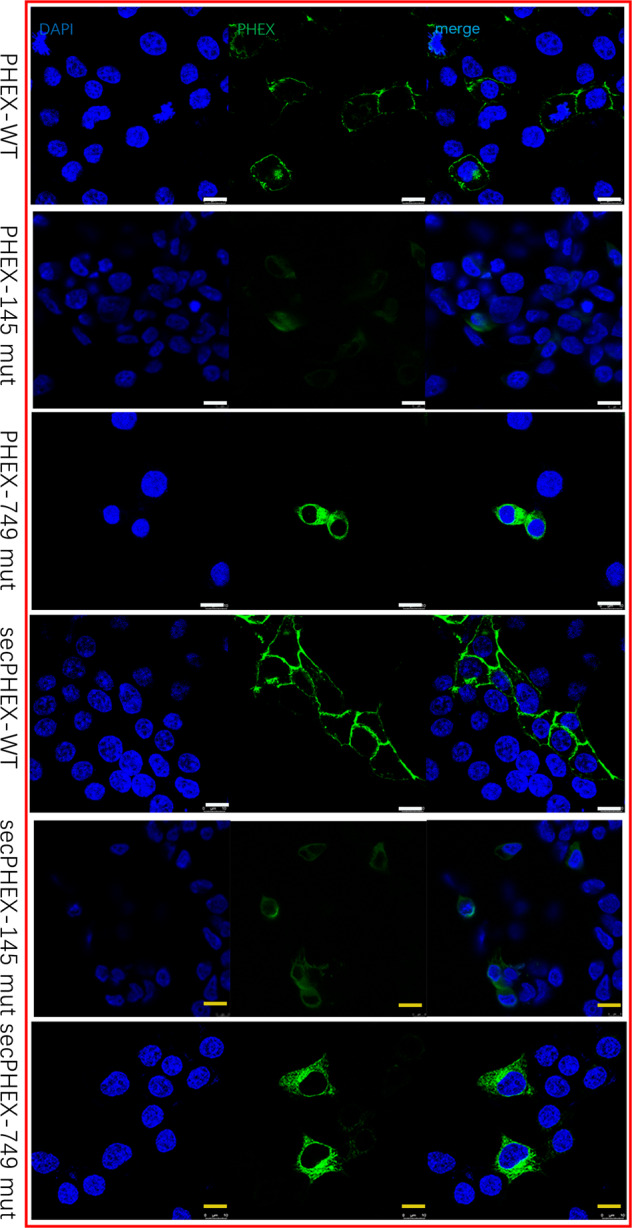
The localization of wild type and mutant PHEX and secPHEX, as detected using immunofluorescence in HEK293T cells single-transfected with *PHEX* and sec*PHEX*. Wild-type PHEX and secPHEX proteins are mainly located in the cell membrane and cytoplasm, while the localization of p.Glu145* and p.Trp749Arg mutated PHEX and secPHEX proteins in the cells changes such that they are widely distributed. At the same time, the expression of mutant PHEX and secPHEX proteins decreases significantly, especially the p.Glu145* mutants. Scale bar, 8 μm.

The packaged *PHEX*-WT, *PHEX*-mut145, *PHEX*-mut749, sec*PHEX*-WT, sec*PHEX*-mut145, and sec*PHEX*-mut749 plasmids were separately transfected into HEK293T cells for 24 h. Cells were then incubated with Flag-PHEX, co-stained with the PHEX protein, secPHEX, and the cell structure was observed using fluorescence microscopy. Wild-type PHEX and secPHEX proteins were mainly located in the cell membrane and cytoplasm, whereas the location of mutant PHEX and secPHEX proteins changed after p.Glu145* and p.Trp749Arg variants, and were widely distributed. The expression of mutant PHEX and secPHEX proteins decreased significantly, especially in the p.Glu145* mutant (Fig. 2).

**Fig. 3:**
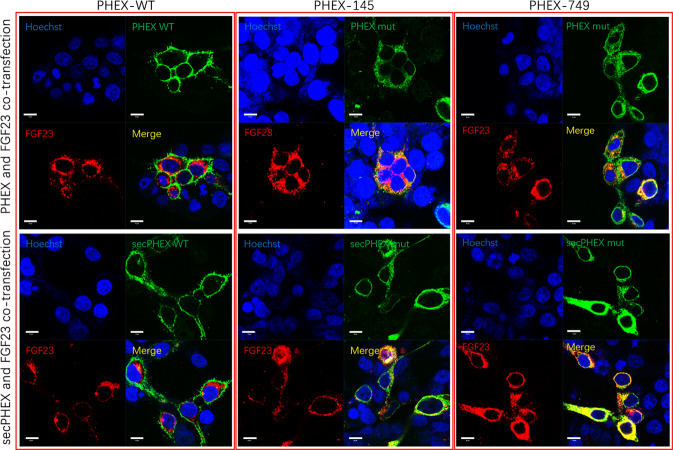
The localization of wild-type and mutant PHEX, secPHEX, and FGF23, as detected using immunofluorescence in HEK293T cells co-transfected with *PHEX*, sec*PHEX*, and *FGF23*. Wild-type PHEX, secPHEX, and FGF23 proteins are distributed in the cell membrane or endoplasmic reticulum, while the mutant is located in the nuclear membrane (possibly endoplasmic reticulum) and cytoplasm. In order to clearly show the positional correlation between co-transfected FGF23 and PHEX expressed in cells, the figures were overexposed. WT wild type, mut mutation type, sec secretory type. Scale bar, 10 μm.

The packaged *PHEX*-WT, *PHEX*-mut145, *PHEX*-mut749, sec*PHEX*-WT, sec*PHEX*-mut145, sec*PHEX*-mut749 plasmids, and the constructed *FGF23* plasmids were co-transfected into HEK293T cells for 24 h. After being fixed and incubated with Flag-PHEX and FGF23 antibodies, the distribution of PHEX and FGF23 proteins in HEK293T cells was observed by fluorescence microscopy. Wild-type PHEX, secPHEX, and FGF23 proteins in the *PHEX*-WT and sec*PHEX*-WT groups were distributed in the cell membrane or endoplasmic reticulum, while the mutant PHEX and secPHEX proteins in the *PHEX*-mut145 and sec*PHEX*-mut145 groups were mostly co-localized with FGF23 in the nucleus, nuclear membrane (possibly endoplasmic reticulum) and cytoplasm (Fig. 3).

### Expression of *PHEX* and *secPHEX* mRNA

**Fig. 4:**
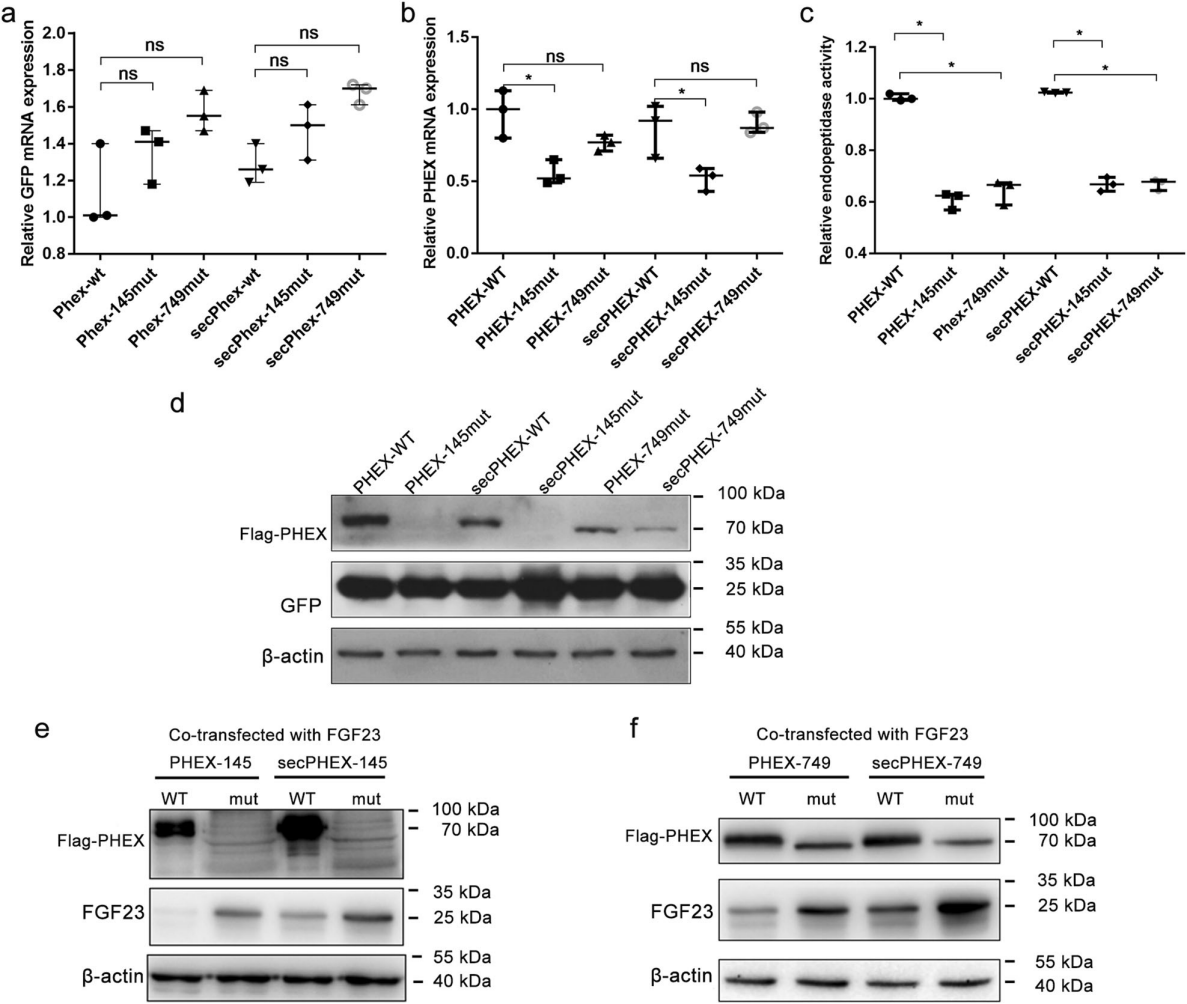
Functional identification of PHEX p.Glu 145* and p.Trp749Arg. **a**, **b** The expression of *PHEX*, sec*PHEX* and green fluorescence protein (*GFP*) in transfected HEK293T cells, as detected by quantitative polymerase chain reaction (qPCR). Under the condition that the internal reference glyceraldehyde 3-phosphate dehydrogenase (*GAPDH*) is relatively consistent among each group, the expression concentration of *GFP* in the external reference are the same. The expression of mRNA in cells decreases by *PHEX* and sec*PHEX* p.Glu145* nonsense mutations (median ± range, *n* = 3, nonparametric tests, *p < 0.05); there is no difference in the expression of mRNA in p.Trp749Arg. **c** p.Glu145* and p.Trp749Arg mutants all lead to the decrease in intracellular PHEX endopeptidase activity (median ± range, *n* = 3, nonparametric tests, *p < 0.05). **d** Western blot (WB) showed that the PHEX and secPHEX proteins mutated by p.Glu145* and p.Trp749Arg are obviously decreased in single-transfected *PHEX* and sec*PHEX* cells. **e**, **f** the expression of PHEX and secPHEX mutant proteins is decreased using WB, while the expression of FGF23 protein increases in *PHEX*, sec*PHEX* and *FGF23* co-transfected cells. β-actin is the internal reference. WT wild type, mut mutation type, sec secretory type, GFP, external reference; GAPDH and β-actin, internal reference.

The expression of *PHEX*, sec*PHEX*, green fluorescence protein (*GFP*), and the internal reference glyceraldehyde 3-phosphate dehydrogenase (*GAPDH*, Fig. 4a, b) was detected using qPCR. Under the condition that the *GAPDH* concentration of the internal reference was relatively consistent, the expression concentration of *GFP* in the external reference were the same, indicating that the level of cell transfer among each group was the same. Using *GFP* as an external parameter, the expression of *PHEX* mRNA in cells was determined. The *PHEX* p.Glu145* nonsense mutation significantly decreased intracellular *PHEX* mRNA expression (*p* < 0.05), and the sec*PHEX* p.Glu145* nonsense mutation caused a decrease in sec*PHEX* mRNA expression (*p* < 0.01). Regarding *PHEX* or sec*PHEX* p.Trp749Arg missense variants, there was no significant difference in *PHEX* or sec*PHEX* mRNA variants in wild-type and mutant cells (*p* > 0.05).

### PHEX endopeptidase activity

The results of the enzyme activity test showed that both *PHEX* p.Glu145* nonsense mutation and p.Trp749Arg missense mutation, led to a decrease in PHEX endopeptidase activity during hydrolysis (Fig. 4c).

### Differences in the expression of PHEX, secPHEX, and FGF23 proteins in HEK293T cells

The expression of PHEX, GFP, and β-actin was detected using western blotting after the *PHEX* and sec*PHEX* plasmids were transfected into HEK293T cells (Fig. 4d). Under the condition where β-actin of the internal reference was relatively consistent among each group, the expression concentration of GFP in the external reference was the same, indicating that the level of cell transfer among each group was the same. Using GFP as an external parameter, the expression of PHEX in the cells was determined. The *PHEX* p.Glu145* nonsense mutation led to a decrease in intracellular PHEX protein expression. The sec*PHEX* p.Glu145* nonsense mutation led to a decrease in secPHEX protein expression, whereas the *PHEX* or sec*PHEX* p.Trp749Arg missense mutation resulted in a decrease in PHEX or secPHEX protein expression. In addition, no expression of FGF23 was detected in the four groups.

After *PHEX* and sec*PHEX* plasmids were co-transfected with *FGF23* plasmids into HEK293T cells, WB analysis showed that the expression of PHEX and secPHEX decreased, and the band size was smaller after the appearance of p.Glu145* and p.Trp749Arg variants in *PHEX* and sec*PHEX*, which promoted the expression of the FGF23 protein, especially the p.Glu145* mutation (Fig. 4e, f). It is possible that the function of the PHEX protein was significantly decreased due to the truncation of the protein caused by the p.Glu145* mutation.

### *PHEX* overexpression and dual-luciferase reporter gene assay system

**Fig. 5:**
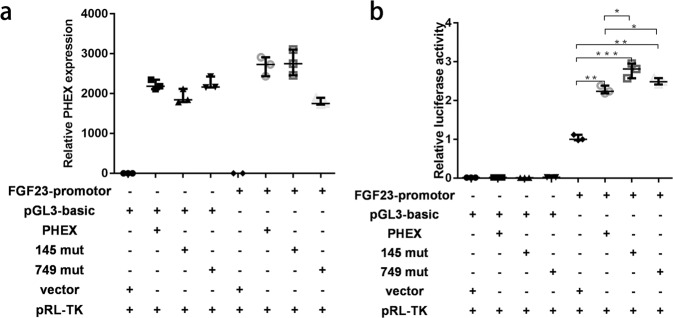
PHEX regulates the FGF23 promoter. **a** RT-qPCR was used to detect the expression of *PHEX* in each group in HEK293T cells. Under the condition that the internal reference β-actin is relatively consistent among each group, the mRNA concentration in the *PHEX* overexpression group was significantly increased. **b** The double luciferase reporter gene method was used to detect *FGF23* promoter activity. With Renilla luciferase as the internal reference, the relative fluorescence intensity calculation formula = firefly luciferase/renilla luciferase. After overexpression of wild-type or mutant *PHEX*, firefly luciferase activity was enhanced. Mut mutation pRL-TK Ranilla luciferase vector plasmid. (median ± range, *n* = 3, nonparametric tests, ^*^p < 0.05; ^**^p< 0.01; ^***^p < 0.001).

Using RT-qPCR to detect *PHEX* expression in 293 T cells, the mRNA concentration of this gene was significantly upregulated in the PHEX overexpression group. Using Renilla luciferase as an internal control (relative fluorescence intensity calculation formula = firefly luciferase/renilla luciferase), after overexpression of wild-type and mutant PHEX, the firefly luciferase activity increased. Compared with wild type, *PHEX* p.Glu145 *And p.Trp749Arg firefly luciferase activity was significantly enhanced (*P* < 0.05) (Fig. 5).

## Discussion

The purpose of this study was to prove the pathogenicity of two variants of the *PHEX* gene found in a previous study [22]. It has been preliminarily confirmed that p.Glu145* and p.Trp749Arg are pathogenic variants, and the molecular mechanism of XLH has been suggested. Interestingly, this study found that PHEX negatively regulates the transcription of FGF23 promoter, which provides a new explanation for the regulatory mechanism between PHEX and FGF23.

PHEX pathogenicity is a complex pathological process. Research shows that truncated proteins produced by some nonsense variants may affect the splice mode of RNA precursors (such as c.436 + 1 G > C), thereby affecting the conformation and function of proteins [30]. Some missense variants (such as p.C85R, p.G579R, and p.P558A) influence the PHEX protein spatial structure and cause the mutated PHEX protein to be isolated within the endoplasmic reticulum; thus, it cannot reach the plasma membrane and influence function and disease [1, 31]. Likewise, the immunofluorescence localization of the two variants we found showed that the mutants were trapped in the endoplasmic reticulum compared to the wild-type localization on the cell membrane. Moreover, the variation of p.Glu145* and p.Trp749Arg resulted in the decrease of PHEX protein expression and enzyme activity. In the C-terminal area, extracellular missense variants affect the formation of disulfide bonds, leading to secondary protein structural defects, and destroy function by inhibiting the enzyme activity of the PHEX protein [32]. Consistent with these research, the p.Trp749Arg mutation occurs near two highly conserved cysteine residues at the C-terminus of *PHEX* gene, which are critical for disulfide bond formation and protein folding. Therefore, a single base change at this position may alter the secondary structure of the PHEX protein and then render it useless. p.Trp749Arg is the substitution of a large positively charged residue for a small residue, which increases the local charge of the *PHEX* gene product and may play a role in different spatial conformations [1]. Thus, the two variants p.Glu145* and p.Trp749Arg lead to the decline of PHEX protein function.

The active form of FGF23 is the full-length protein from the 25th to 251st amino acids (227 amino acids in total) after the transcription of *FGF23* and fragmentation from position 24. Under physiological conditions, the hydrolysis of Arg176-R-R-Arg179 leads to the non-active form of FGF23. During secretion in HEK293 and COS-7 cells, FGF23 is processed by a subtilisin-like proprotein convertase (SPC) cleavage site (RXXR motif) at the C-terminal of amino acids 179–180, and the expression of FGF23 cannot be detected in cells [33]. FGF23 protein expression was not detected in HEK293T cells after *PHEX* and sec*PHEX* overexpression vectors were separately transfected. Therefore, the *FGF23* overexpression vector and the *PHEX* and sec*PHEX* overexpression vectors were selected for co-transfer into HEK293T cells. There was a negative correlation between PHEX protein and *FGF23*. In addition, after the PHEX protein lost its transmembrane binding domain (secPHEX protein), the ability to regulate *FGF23* was further decreased.

FGF23 plays an important role in the kidney and bone metabolism. Hypophosphatemia caused by FGF23 elevation is the main pathophysiological mechanism of FGF23 and XLH. The expression of FGF23 is regulated mainly by serum phosphate and ossified triol. The increase in serum FGF23 induced by phosphate mainly occurs in bone, which may be related to nicotinamide adenine dinucleotide phosphate (NADPH)-induced reactive oxygen species (ROS) and the mitogen-activated protein kinase kinase (MEK)-ERK pathway [34]. In this study, PHEX and FGF23 were co-transfected into HEK293T cells, and it was found that after PHEX mutation, the expression of FGF23 increased; and the PHEX mutant protein and FGF23 co-localized in the cytoplasm, indicating that there is a certain relationship between PHEX and FGF23. For example, the osteocyte lacunae of Hyp mice are abnormal and enlarged, while treatment with FGF23 antibody can reduce the size of the osteocyte lacunae and improve the tubular tissue [35].

One of the mechanisms by which full-length PHEX regulates serum FGF23 may be through the direct lysis of proprotein convertase subtilisin/kexin type 9 (PC2), and PC2 can be upregulated by PHEX [36]. PC2 promotes the formation of the PHEX-DMP1-integrin complex, which inhibits FGF23 [37] when PC2 and its chaperone, neuroendocrine polypeptide 7B2 (7B2 PC2), are activated. However, there is no possibility of direct interaction between 7B2 PC2 and FGF23; therefore, it remains unclear as to whether PHEX directly regulates FGF23 [38]. In studies affecting FGF23 transcription, HIF-1α and FGF23 were found to co-localize in spindle cells near blood vessels [39]. HIF-1α may be a direct transcriptional activator of FGF23, affecting the expression of FGF23 [39]. At the same time, iron deficiency and inflammation also affect the expression and fragmentation of FGF23 [40]. And we discovered a new regulatory mechanism between *PHEX* and *FGF23*. This suggests that PHEX may mediate hypophosphatemia by regulating *FGF23* promoter to regulate FGF23 concentration. It is revealed that *PHEX* is a direct transcriptional inhibitor of *FGF23* and affects the expression of *FGF23*.

In this study, we have identified two new variants in the Chinese population, along with their cellular function, and tried to clarify the regulatory mechanism between PHEX and FGF23. In addition, the clinical phenotype-gene mutation association analysis of these variants is consistent (as indicated by the case report study). Additionally, different types of variants were compared. These data provide a reference for the molecular genetic pathogenesis of XLH. However, due to certain limitations, we have not replicated the disease phenotype at the animal level, nor have we explored the specific mechanism underlying PHEX induction of FGF23 expression concentration, which we intend to explore in future studies.

## Materials and methods

### Construction and identification of plasmids

The plasmid synthesis scheme is as follows (Fig. 1b, d) using the expression vector.

**Fig. 6:**
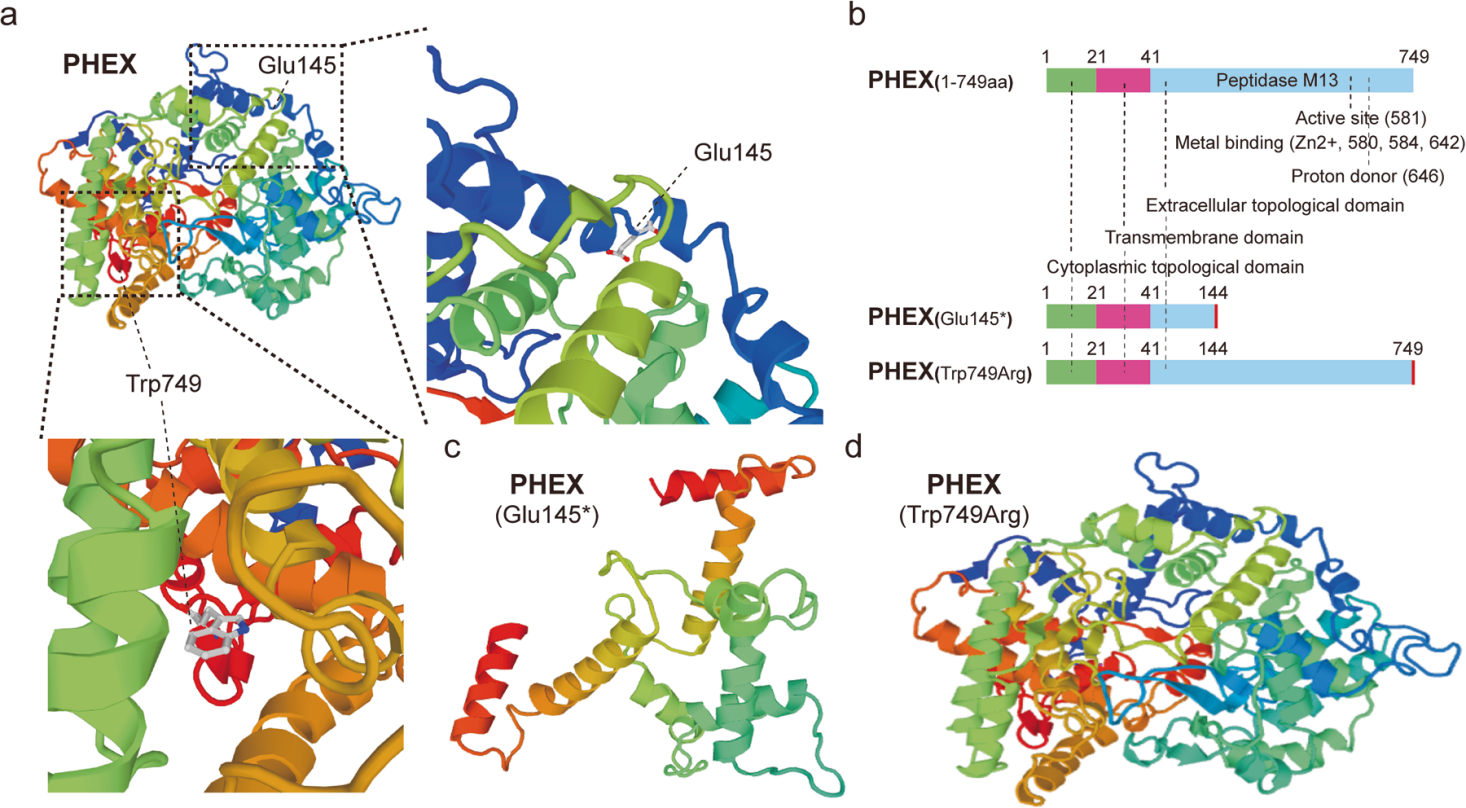
The structure prediction map of PHEX protein mutant. **a** three-dimensional crystal structure map of PHEX protein and the location of mutation sites (dmam SwissMe model database, URL: https://swissmodel.expasy.org/repository/uniprot/P78562). **b** the functional domain map of PHEX protein and its variants. **c**, **d** prediction and comparison of the three-dimensional structure of normal PHEX and Glu145* mutation and Trp749Arg mutation using Swiss-model. (URL: https://swissmodel.expasy.org/repository/uniprot/P78562).

pBoBi-N-3*Flag and pGL3 basic. To synthesize the *PHEX* gene, the insert size was 2262 bp (see Supplement 1 for the sequence), and the restriction site used the restriction enzymes XbaI-BsrGI. The following mutant plasmids were constructed: Construct mutant plasmid 1: pBoBi-N-3*Flag-secPHEX, where secPHEX was derived from amino acids 109–120. For the deletion mutation of *PHEX*, the insert size was 2250 bp (see Supplement 2), and the restriction site was XbaI-AgeI. To construct mutant plasmid 2, the pBoBi-N-3*Flag-PHEX-mut145, the 433 G > T(p.Glu145*) mutation of *PHEX* was introduced. To construct mutant plasmid 3, the pBoBi-N-3*Flag-PHEX-mut749, the 2245 T > C(p.Trp749Arg) mutation of *PHEX* was introduced. To construct mutant plasmid 4, the pBoBi-N-3*Flag-secPHEX-mut145 was constructed using the pBoBi-N-3*Flag-secPHEX plasmid, and the 421 G > T mutation of sec*PHEX* was introduced. To construct mutant plasmid 5, pBoBi-N-3*Flag-secPHEX-mut749 was constructed using the pBoBi-N-3*Flag-secPHEX plasmid, and the 2233 T > C mutation of sec*PHEX* was introduced. Construct plasmid 6: pGL3 basic-*FGF23* containing *FGF23* promoter sequence, and the restriction site was KpnI-HindIII (see Supplement 3). The target gene was amplified and digested with DpnI, and the digested product was transformed into *Escherichia coli* DH5a. Colonies were screened with antibiotics and monoclonal colonies were selected, and sequencing identification was conducted. The *PHEX*-WT, *PHEX*-mut, sec*PHEX*-WT, sec*PHEX*-mut gene cloning, and related PCR primers were synthesized by General Biosystems (Anhui, China). *FGF23* gene synthesis and plasmid construction were performed by Shanghai Generay Biotech Co., Ltd. (Shanghai, China). *FGF23* was 765 bp in length and was cloned into pBOBI-cs2-c-3HA using the BamHI/XhoI restriction site. A schematic diagram of the PHEX protein and the different fragments is shown in Fig. 6.

### Cell culture, transfection, expression level, and functional identification

HEK293T cells were cultured in Dulbecco’s modified Eagle medium containing high amount of glucose, 10% fetal bovine serum, and 1% penicillin/streptomycin. HEK293T cells were digested with trypsin and seeded into 24-well plates. When the cells reached 50%–60% confluency, the single *PHEX* gene and the co-expression plasmid of *PHEX* and *FGF23* were used for transformation. The groups were as follows: (1) *PHEX*-WT + pLKO-*GFP*, (2) *PHEX*-mut145 + pLKO-*GFP*, (3) sec*PHEX*-WT + pLKO-*GFP*, (4) sec*PHEX*-mut145 + pLKO-*GFP*, (5) *PHEX*-mut749 + pLKO-*GFP*, and (6) sec*PHEX*-mut749 + pLKO-*GFP*. The HEK293T cell line was provided by Life Technology Co., Ltd. (Xiamen, China). Carry out the plasmid transfection of the dual-luciferase reporter gene experiment. The cells were co-transfected with the reporter gene plasmid and phRL-TK (internal reference) and divided into 8 groups: (1) pGL3-basic+pLV; (2) pGL3-basic+*PHEX*; (3) pGL3-basic+mut145; (4) pGL3-basic+mut749; (5) *FGF23*-promotor+pLV; (6) *FGF23*-promotor+*PHEX*; (7) *FGF23*-promotor+mut145; (8) *FGF23*-promotor+mut749, and follow the instructions for TurboFect (R0531, Thermo, Waltham, USA) to operate.

### Detection of PHEX protein and FGF23 protein expression by western blot (WB)

HEK293T cell total protein was extracted by culture lysis, and the concentration of PHEX protein was detected. Protein samples and protein markers were added to 10% electrophoresis gel in the desired order. After electrophoresis, proteins were transferred to a polyvinylidene difluoride membrane, soaked in 5% skim milk for 1 h before the primary antibody (rabbit anti-Flag (Proteintec, 20543–1-AP), 1:1000; mouse anti FGF23 (Abcam, Ab190702), 1:50; anti-GFP antiboday (CST, 2956 S); β-actin antibody (Santa Cruz Biotechnology, SC-47778)) solution was added and incubated at 4 °C overnight. After washing the membrane three times, the secondary antibody (goat anti-rabbit/mouse (Gibco, A-11034/A-11032), 1:5000) was added for 1 h. After washing three times, the membranes were exposed.

### Detection of *PHEX* mRNA expression by RT-PCR

Cellular RNA was extracted according to the instructions of the Animal Total RNA Rapid Extraction Kit, and the first chain of cDNA was synthesized according to the instructions of the 5× All-In-One RT Master Mix. Primer 3 software (version 0.4.0, San Francisco, USA) was used to design *PHEX-*, *eGFP-*, and *GAPDH*-specific primers, as follows (5'-3'): h*PHEX* (F: CAGGCATCACATTCACCAAC, R: GCCTCTGTTCATCGTGGAAT), *GFP* (F: ACGTAAACGGCCACAAGTTC, R: AAGTCGTGCTGCTTCATGTG); *GAPDH* (F: CAAGGTCATCCATGACAACTTTG, R: GTCCA CCACCCTGTTGCTGTAG). The qRT reaction system was 20 μL, and each cell sample was duplicated three times using RT-PCR.

### Dual-luciferase reporter assay system

After transfection for 48 h, cells were collected and rinsed with pre-cooled PBS. 5×PLB was diluted with deionized water to 1×PLB, which was balanced to room temperature before use. Add 100 μL of diluted 1×PLB to each well, and shake for 15 min at room temperature on a shaker to perform lysis. Add 20 μL of the above cell lysate and 100 μL of pre-mixed LAR II to each well of the 96-well white opaque ELISA plate, and measure the data after 2 s. Add 100 μL pre-mixed Stop&Glo Reagent to each well, and measure the data after standing for 2 s.

### Cellular immunofluorescence

After the transfected HEK293T cells were fixed, permeabilized, and blocked, the primary antibody (Flag, 1:100 (sigma, F2555); FGF23, 1:200(Abcam, Ab190702)) was added and incubated overnight at 4 °C. The next day, cells were washed with phosphate-buffered saline (PBS) three times, and the fluorescent secondary antibody (1:500, Flag488(Gibco, A-11034), FGF23–594(Gibco, A-11032)) was added and incubated for 1 h at room temperature. The nuclei were stained with Hoechst (1:1000) and incubated at room temperature for 20 min in the dark. The cells were washed with PBS three times, incubated with 4,6-diamidino-2-phenylindole at room temperature for 5 min, mounted with 50% glycerol, and photographed using a laser confocal microscope (LSM780, Carl Zeiss AG, Jena, Germany).

### Determination of PHEX protein activity

The cultured HEK293T cells were collected and lysed in radioimmunoprecipitation assay buffer, and the supernatant of the lysate was used for enzyme activity determination. For the enzyme activity assay (Varioskan LUX, Thermo Scientific, Waltham, US), the polypeptide Abz-GFSDYK (Dnp)-OH (10 μM), and 100 μg total protein were mixed and incubated at 37 °C for 30 min. The absorbance was measured using a microplate reader at an excitation wavelength of 420 nm and an emission wavelength of 320 nm.

### Statistical method

Data were statistically analyzed using IBM SPSS Statistics 22, and values were expressed as Median with range. Median of multiple groups were compared using independent sample nonparametric tests. *P*-value of <0.05 was considered statistically significant.

## Supplementary information

checklist

Original Data File

Supplement 1

Supplement 2

Supplement 3

Supplementary file legends

## Data Availability

Data supporting the findings of this study are available from the corresponding author upon reasonable request.
