# Supplementary figures and images for: Function of PHEX mutations p.Glu145* and p.Trp749Arg in families with X-linked hypophosphatemic rickets by the negative regulation mechanism on FGF23 promoter transcription
Source: Cell Death Dis. 2022 Jun 2;13(6):518. doi: 10.1038/s41419-022-04969-5 (PMC9163062; doi:10.1038/s41419-022-04969-5)

A

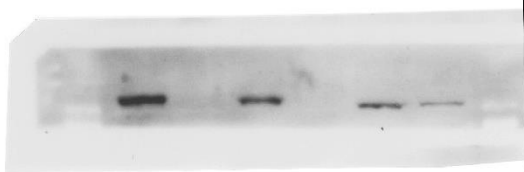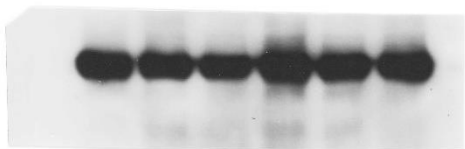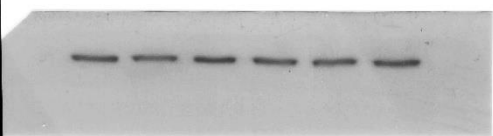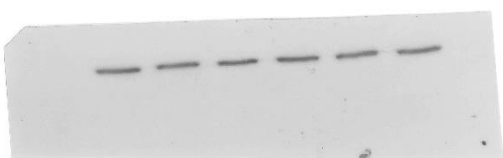

**B**

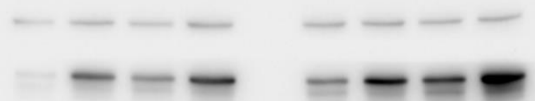

**C**

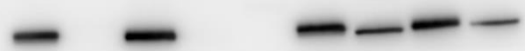

**D**

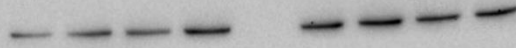

**F**

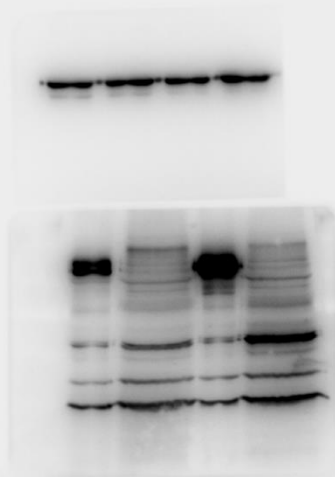

Supplement: Supplementary file 2 — Original Data File [file 41419_2022_4969_MOESM2_ESM.pdf]

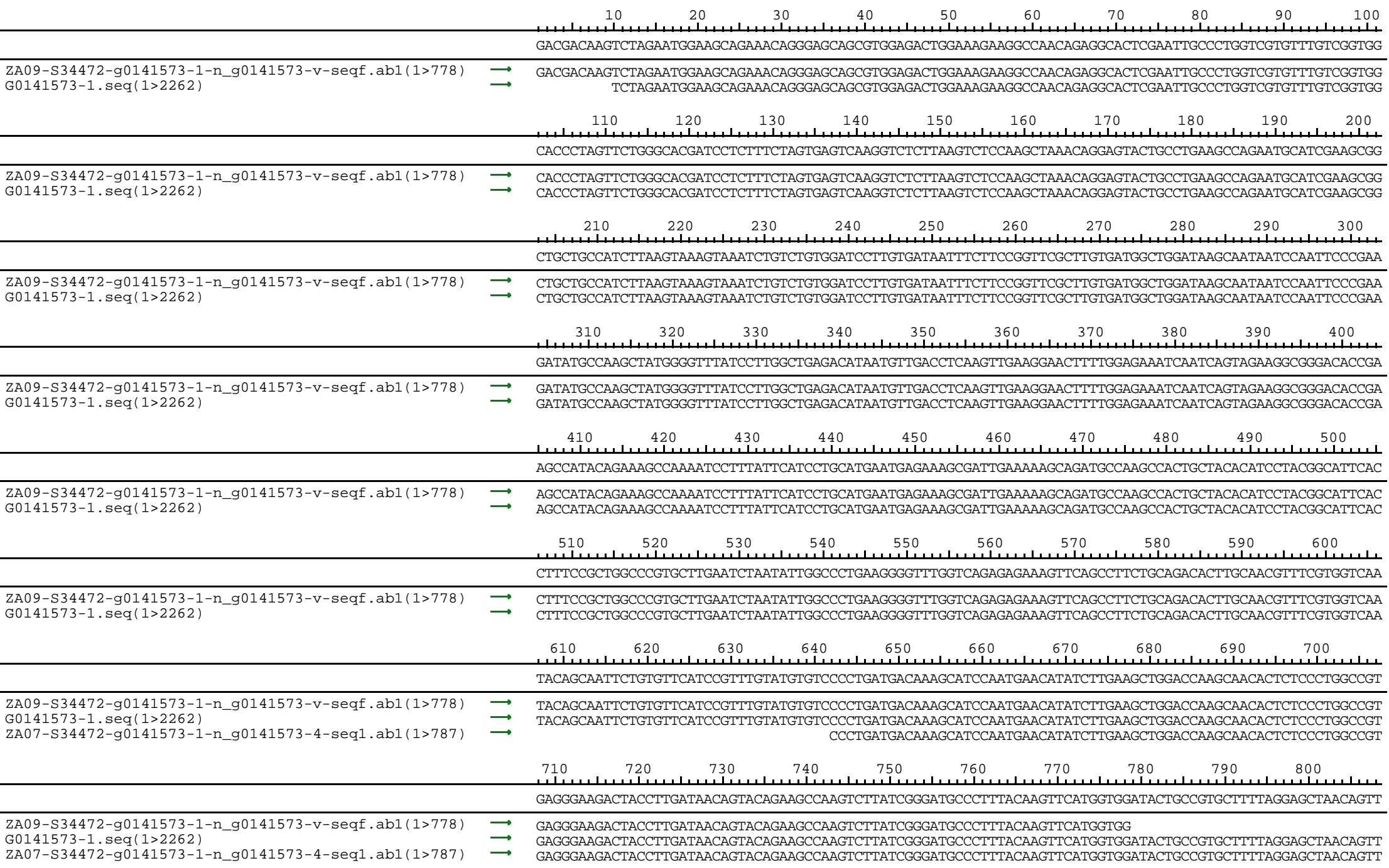

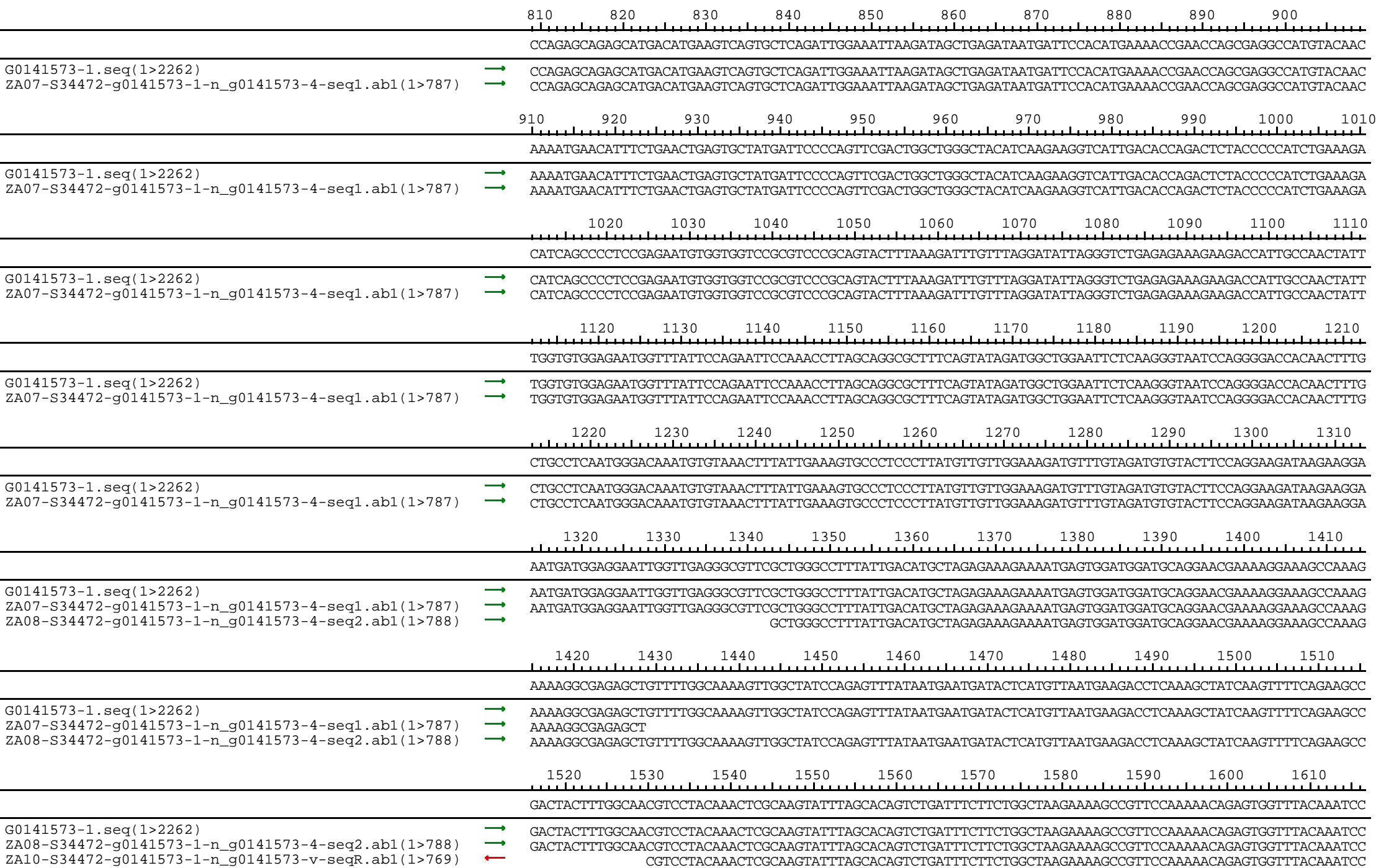

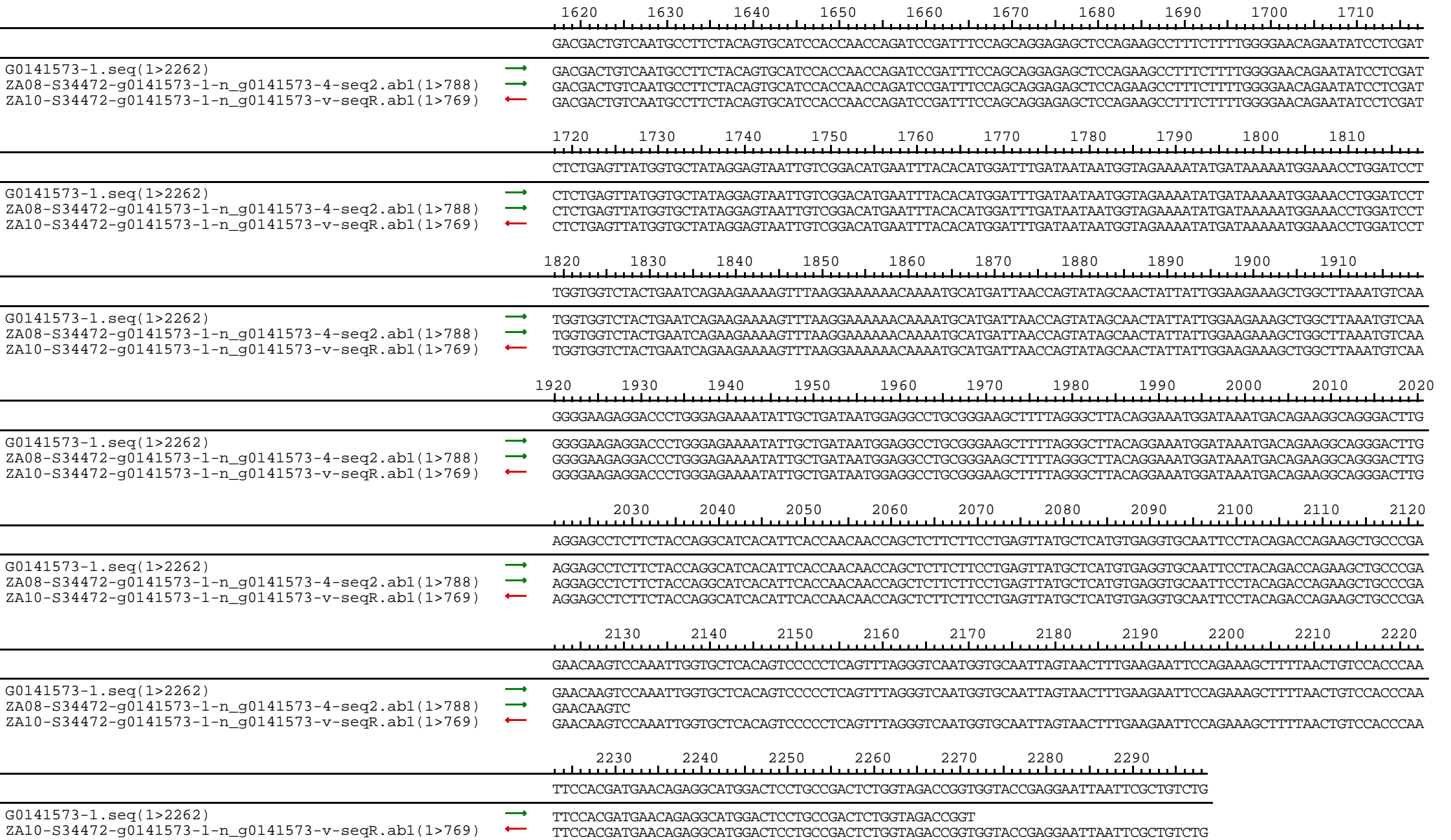

Supplement: Supplementary file 3 — Supplement 1 [file 41419_2022_4969_MOESM3_ESM.pdf]

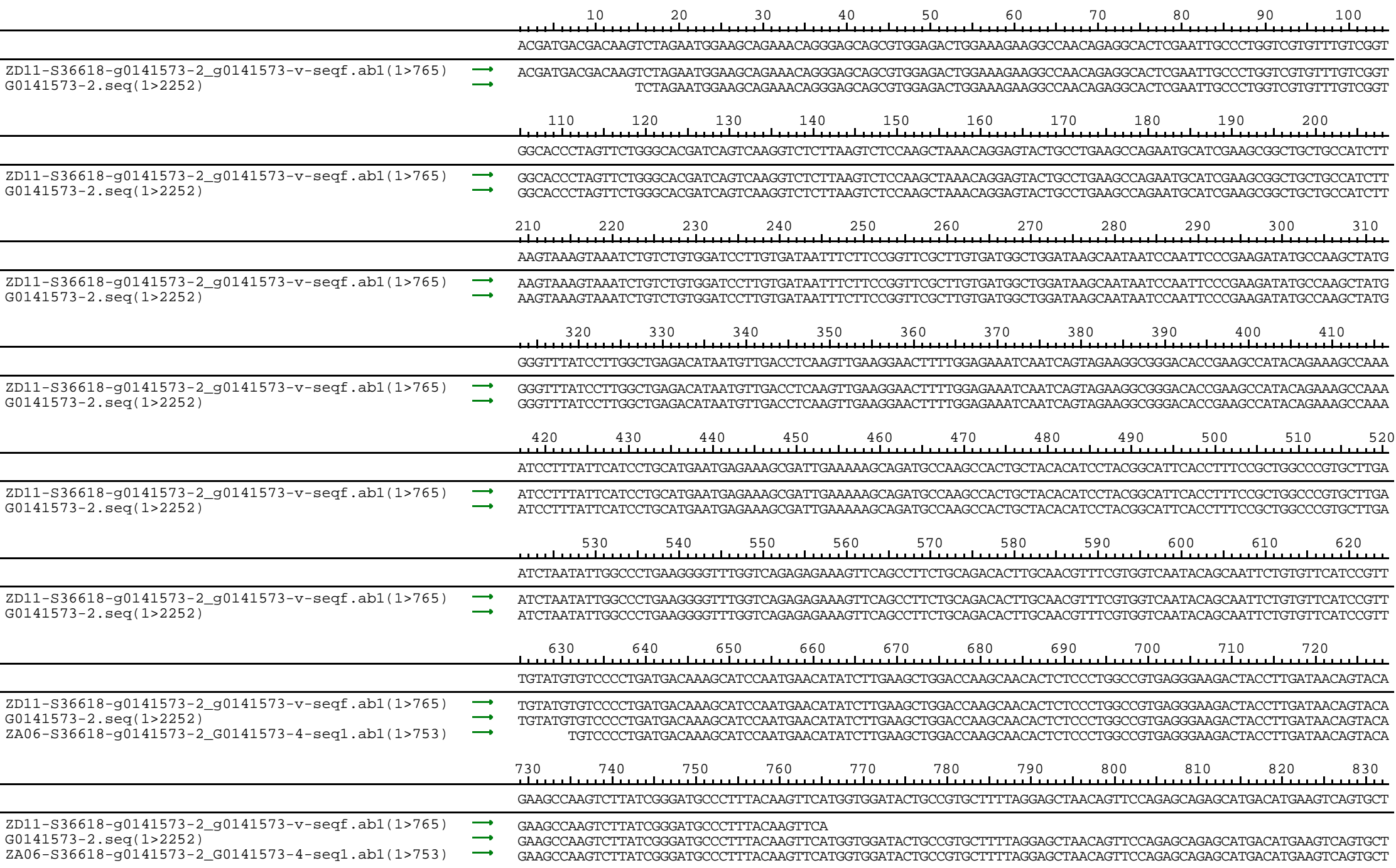

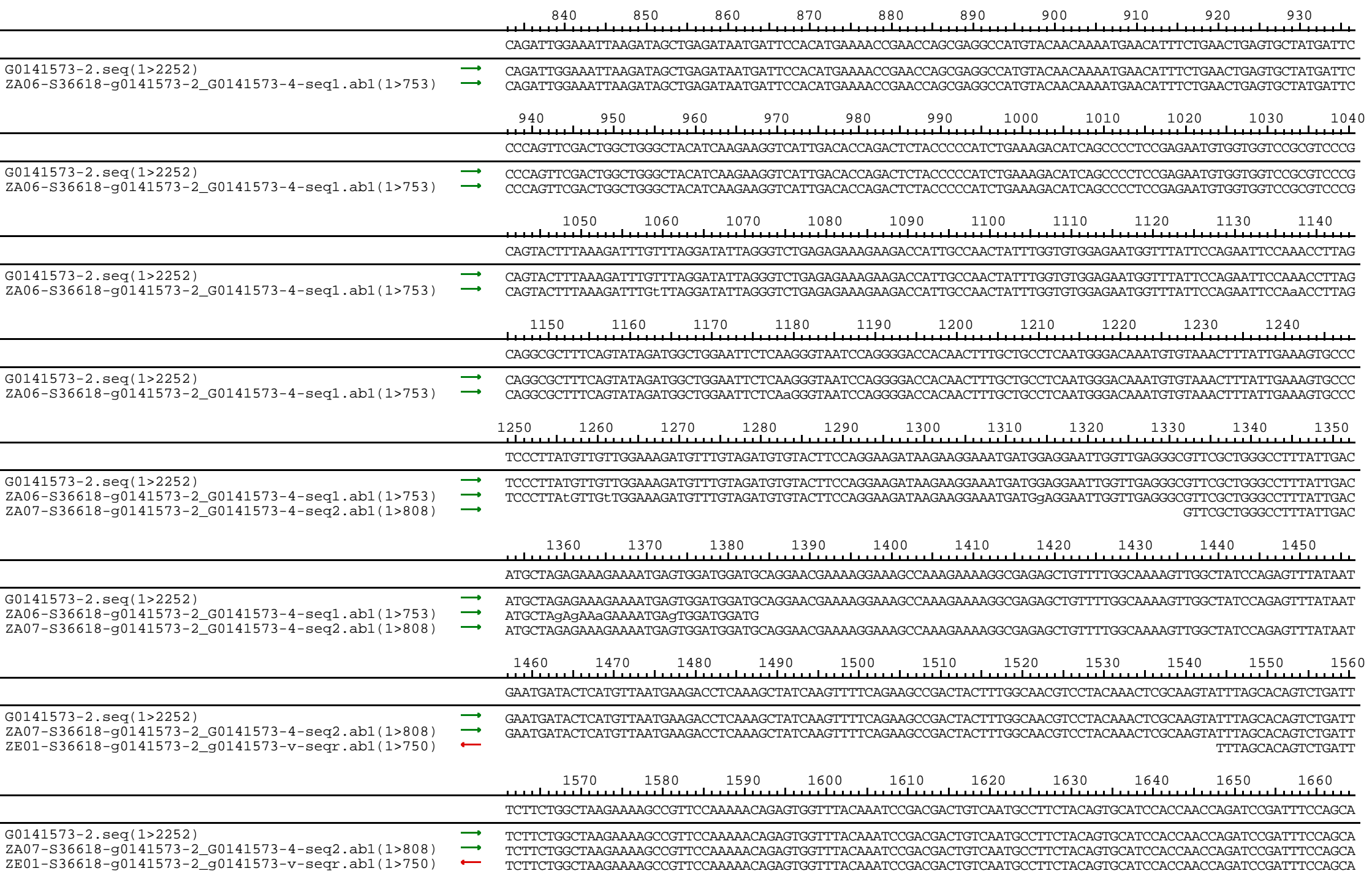

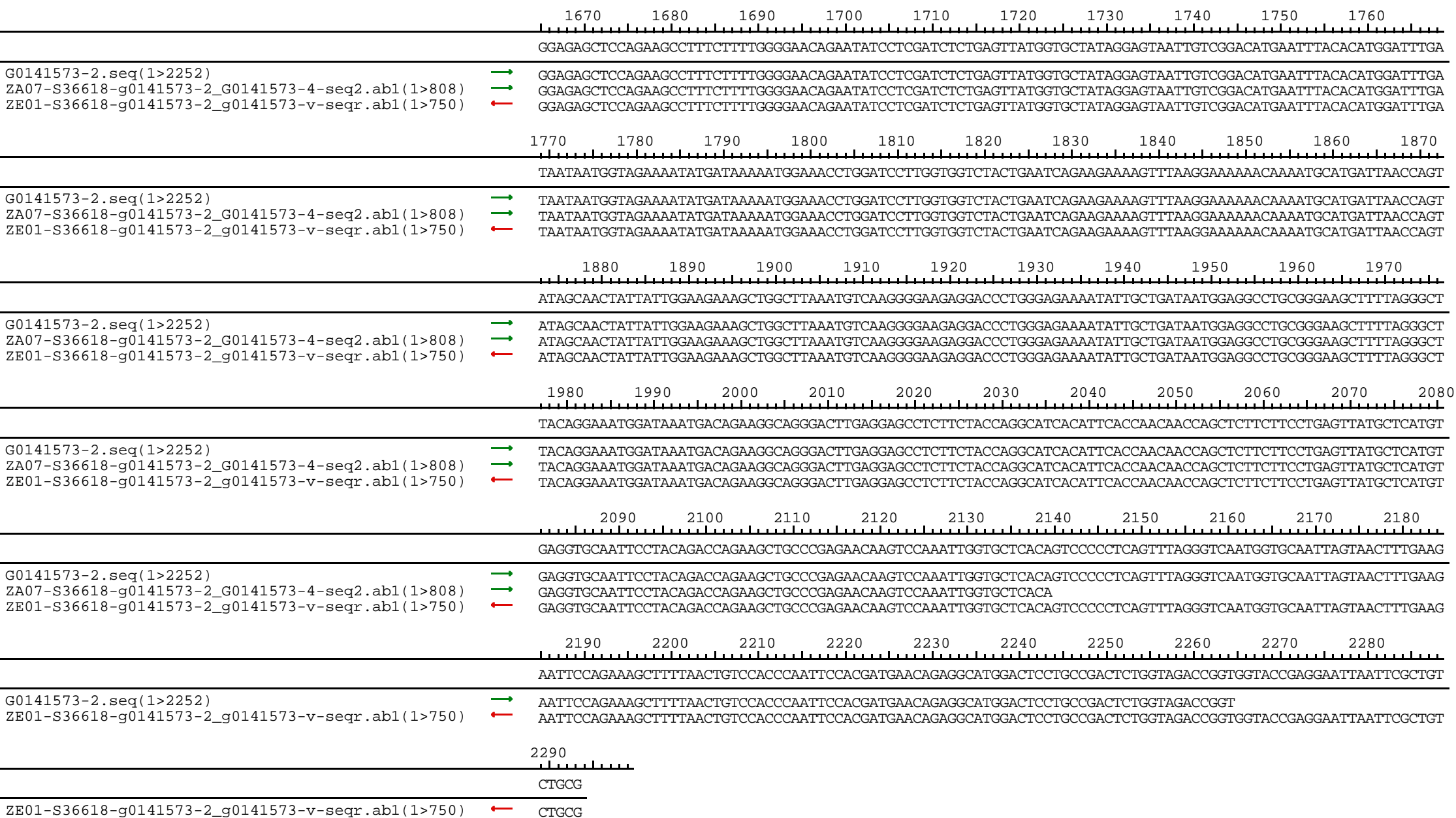

Supplement: Supplementary file 4 — Supplement 2 [file 41419_2022_4969_MOESM4_ESM.pdf]
