# Supplementary material for: Function of PHEX mutations p.Glu145* and p.Trp749Arg in families with X-linked hypophosphatemic rickets by the negative regulation mechanism on FGF23 promoter transcription
Source: Cell Death Dis. 2022 Jun 2;13(6):518. doi: 10.1038/s41419-022-04969-5 (PMC9163062; doi:10.1038/s41419-022-04969-5)
Supplement: Supplementary file 5 — Supplement 3 [file 41419_2022_4969_MOESM5_ESM.pdf]

A

TspDTI
BspCNI PspFI CviKI-1
KpnI Hpy188III
BseYI
BseMII MnlI
AluI
RsaI CviKI-1 HpyCH4III
NlaIV **XbaI**
SetI BfaI
CviQI HphI **BfrBI** MseI Hpy188III
BanI BsaXI SetI BtsCI MluCI MseI
Acc65I DdeI SfcI HpyCH4V Hpy188III MaeIII Tsp45I FokI BstF5I HpyCH4V **NsiI** Tsp509I SfaNI SspI

5' GGTACCcagcctcctgagtagctatgactacaggtgcatatcaccactcctgacttcttattgtgagtatttagtggtcactttggggattacaatatgcatccttaatttatcacagtctagagttaatattgtaagatttttacata 150
3' CCATGGgtcggaggactcatcgatactgatgtccacgtatagtggtgaggactgaagaataacactcataaatcaccagtgaaacccctaatgttatacgtaggaattaaatagtgtcagatctcaattataacattctaaaaatgtat

HpyCH4III
BfuAI TspRI TseI
BspMI BtsIMutI ApeKI
HpyCH4V PsiI MseI Fnu4HI
TspRI MaeIII BtsCI MluCI **AflII** BlsI
HpyCH4III BtsIMutI TspDTI FokI TsoI BstF5I MnlI BccI MaeIII SetI Tsp509I **SmlI** CviKI-1 BbvI BarI HpyAV

cagtgttatcatcttgtaaccatattgtttcatttatccctcccatcctatgtgggagttttgccttatatattacatctatatatgttacaaaacctgcatacagtgttataattttgccttaagcagcctgttagtgaaatcaggga 300
gtcacaatagtagaacattggtataacaaagtaaatagggagggtaggatacaccctcaaaacggaatatataatgtagatatatacaatgttttggacgtatgtcacaatattaaaacggaattcgtcggacaatcactttagtccct

BstZ17I MluCI
AccI Hpy166II MseI MluCI BccI CviAII MluCI Tsp509I HpyAV
SetI MboII BarI DraI BccI MseI Tsp509I MaeIII FatI NlaIII Tsp509I MseI BccI

agaaggtatacaatatatatagtttaaatgtattcacacatttaatgatggtttctttctgtaattctaaatgtccatctggtgttactcctttttcagcatggcaataatttctctccatttttattccatcttaattttgaaggacat 450
tcttccatatgttatatatatcaaatttacataagtgtgtaaattactaccaaaagaaagacattaagatttacaggtagaccacaatgaggaaaaagtcgtaccgttattaaagagaggtaaaaataaggtagaattaaaacttcctgta

BtgI CviAII
FatI Hin4I
AluI **StyI** NlaIII AluI
MluCI CviKI-1 **BbsI** BfaI **NcoI** CviKI-1
Tsp509I SetI SetI SetI MboII TsoI BsaJI MslI MboII Hin4I SetI MnlI AccI Hpy166II

ttttgctgtatgtgtaattctgggtaggtagctttttatttttctttcaaccttttcaatacatcattccattgtcttctagtttccatggttatgagaagaaatccactactattatagctgttcctctatatataaagtgtcatttgtc 600
aaaacgacatacacattaagacccatccatcgaaaaataaaaagaaagttggaaaagttatgtagtaaggtaacagaagatcaaaggtaccaatactcttctttaggtgatgataatatcgacaaggagatatatatttcacagtaaacag

BfuCI
MboI*
DpnII*
Sau3AI DpnI* MluCI ApoI
HpyCH4III AluI HpyCH4III BstKTI* BspCNI Hpy188I AluI CviQI RsaI Tsp509I MluCI
CviKI-1 BseMII CviKI-1 BslI Tsp509I
SfcI SetI Hpy188I MseI HpyCH4III HpyCH4V MslI HpyCH4V DdeI SetI **TatI** Hpy166II MseI PflMI TsoI

tacagtagctttcagatttctgttgatttttgatttttaacagtttgactgcaatatacatataactgtgatctttgcatttgtcttgcttggagtttctgagcttcttgtattagtacacttatttttaaccaataattgggaaatttt 750
atgtcatcgaaagtctaaagacaactaaaaactaaaaattgtcaaactgacgttatatgtatattgacactagaaacgtaaacagaacgaacctcaaagactcgaagaacataatcatgtgaataaaaattggttattaaccctttaaaa

BfuCI
BsaI MboI*
BcoDI DpnII*
MmeI BsmFI BsmAI Sau3AI MnlI
BsiHKAI PleI HinfI FaqI AluI SetI DpnI*
CviKI-1 MboII SspI BmrI BsrI Bsp1286I MlyI HpyAV NdeI CviKI-1 MseI SetI BstKTI* SetI MboII MaeIII

cagccaccatatcttcaaatatttttactccccagttctttgtgctcctttccttctgggactcaacttactcatatgttggacagcttaataaggtctcacagatcattgaggttctactcatttcaaaaaacttatttctctctg 900
gtcggtggtatagaagtttataaaaatgaggggtcaagaaacacgaggaaaggaagaccctgagttgaatgagtatacaacctgtcgaattattccagagtgtctagtaactccaagatgagtaaaagttttttgaataaagaagagac

FatI
PciI
BfuCI AflIII
MboI* **BaeGI**
DpnII* DpnI* BsiHKAI
Sau3AI BstKTI* Bsp1286I
BseMII HphI* HpyCH4V **Bme1580I**
BspCNI SetI SetI MaeIII Hpy166II CviAII
Hpy188I MluCI AluI **SpeI** Tsp45I HpyCH4V NspI CviAII NlaIII
DdeI Tsp509I CviKI-1 BfaI BfaI Hpy188III **ApaLI** NlaIII MboII FatI BsrI **BciVI** FatI CviAII

ttactcagataagataatttatattagctctatctttagatcaccttactagttctagtgactgcatttctcttgattgtgcacatgttttttcctatttcttctcatgtccagttattttactattactgattgtgtaggatacca 1050
aatgagtctattctattaaatataatcgagatagaaatctagtggaatgatcaagatcactgacgtaaaagagaactaacacgtgtacaaaaaaaggataaagaagagtacaggtcaataaaatgataatgactaacacatcctatggt

BstYI
TfiI FatI MnlI BfuCI BssKI*
HinfI MboII DraI SetI MboI* DpnI* PspGI* BstNI MluCI ApoI
Hpy188I CviAII **EcoNI** MseI DpnII* StyD4I* ScrFI* Tsp509I MluCI
NlaIII MseI NlaIII MseI EarI BslI MboII EarI AciI MaeIII AlwI* Sau3AI BstKTI* DdeI Tsp509I

tgtattaagtctgaatcatgtcctcttcctttaaaaggtgttaagttctcttctggcggtcagttacattattggtagatccttgtagtttgccaggattggtataattctcacttagagcagtctgtttcagtttgaatttagtttt 1200
acataattcagacttagtacaggagaaggaaattttccacaattcaagagaagaccgccagtcaatgtaataaccatctaggaacatcaaacggtcctaaccatattaagagtgaatctcgtcagacaaagtcaaacttaaatcaaaa

BsaI
BcoDI EarI
BsmAI BsaJI
BseMII BssKI*
BspCNI PspGI*
SetI StyD4I*
HphI **AleI**
MboII BstNI
DdeI MslI
BarI ScrFI*
Hpy188I SetI
MnlI BslI
BarI HpyCH4V
BglI CviKI-1 BspCNI ApoI AflIII EcoP15I
MwoI DdeI BseMII MluCI HpyCH4IV SetI EcoP15I
BslI MnlI PsiI CviKI-1 DdeI Tsp509I TaqII TaiI **BsrDI** SfaNI BbvI

aggatataccccctgcctctggggcttagtcttataagaacagccttttgagttctgaggcactcagtaaggtctcttcacccaggtgtggttgaattctaacgtgtttcaacattgcatagcatctggtatcactcactatctatctc 1350
tcctatatggggggacggagaccccgaatcagaatattcttgtcggaaaactcaagactccgtgagtcattccagagaagtgggtccacaccaacttaaagattgcacaaagttgtaacgtatcgtagaccatagtgagtgatagatagag

SetI FatI **AvaII**
HpyCH4V **PpuMI**
PleI CviAII **Sau96I**
AarI Fnu4HI MlyI MnlI **EcoO109I**
TseI BlsI **BstEII** HphI NspI BslI **BmgT120I** CviAII NlaIII Hpy188I
ApeKI SfcI MaeIII HphI NlaIII PflMI SfcI FatI TstI BcoDI
BfuAI MwoI Tsp45I HinfI HpyCH4III BssSI BmrI BsrI HpyAV SetI **BsmI** NdeI TspDTI BsmAI HpyAV HpyAV
BspMI SetI SetI TstI

ctgctgctgtagcaggtgacctttgtaggactcaccgtatcttacctcgtgcatgtgctacccagtctttggcgaaggacctgtagtgaatgctcatgcccatatgtaaagtctctcctttcatagttccttctgaaagaaggaactct 1500
gacgacgacatcgtccactggaaacatcctgagtggcatagaatggagcacgtacacgatgggtcagaaaccgcttcctggacatcacttacgagtacgggtatacatttcagagaggaaaagtatcaaggaagactttcttccttgaga

BcoDI TfiI BsmFI SetI
BsmAI HinfI HpyCH4III BspCNI Hpy166II **BmgBI** TaiI AjuI
Hin4I TspDTI BspCNI MaeIII BtsIMutI FaqI BaeI DdeI HpyCH4IV BccI BaeI
BsrI DdeI Hpy188I BseMII Hin4I HpyCH4III TspRI HpyCH4V BseMII

gggaacaccagtctcagaatgtagattcattatcttggtgtcctgctatcaaatctcacgggacggtaacactgtgtatgtgtttagtgcatagcatttggttactgagatactgacgtggttgtgatggtgtatttccaaataaa 1650
cccttgtggtcagagtcttacatctaagtaatagaaccacaggacgatagtttagagtgcctgccattgtgacacatacacaaatcacgtatcgtaaaccaatgactctatgactgcaccaacactaccacataaaggtttattt

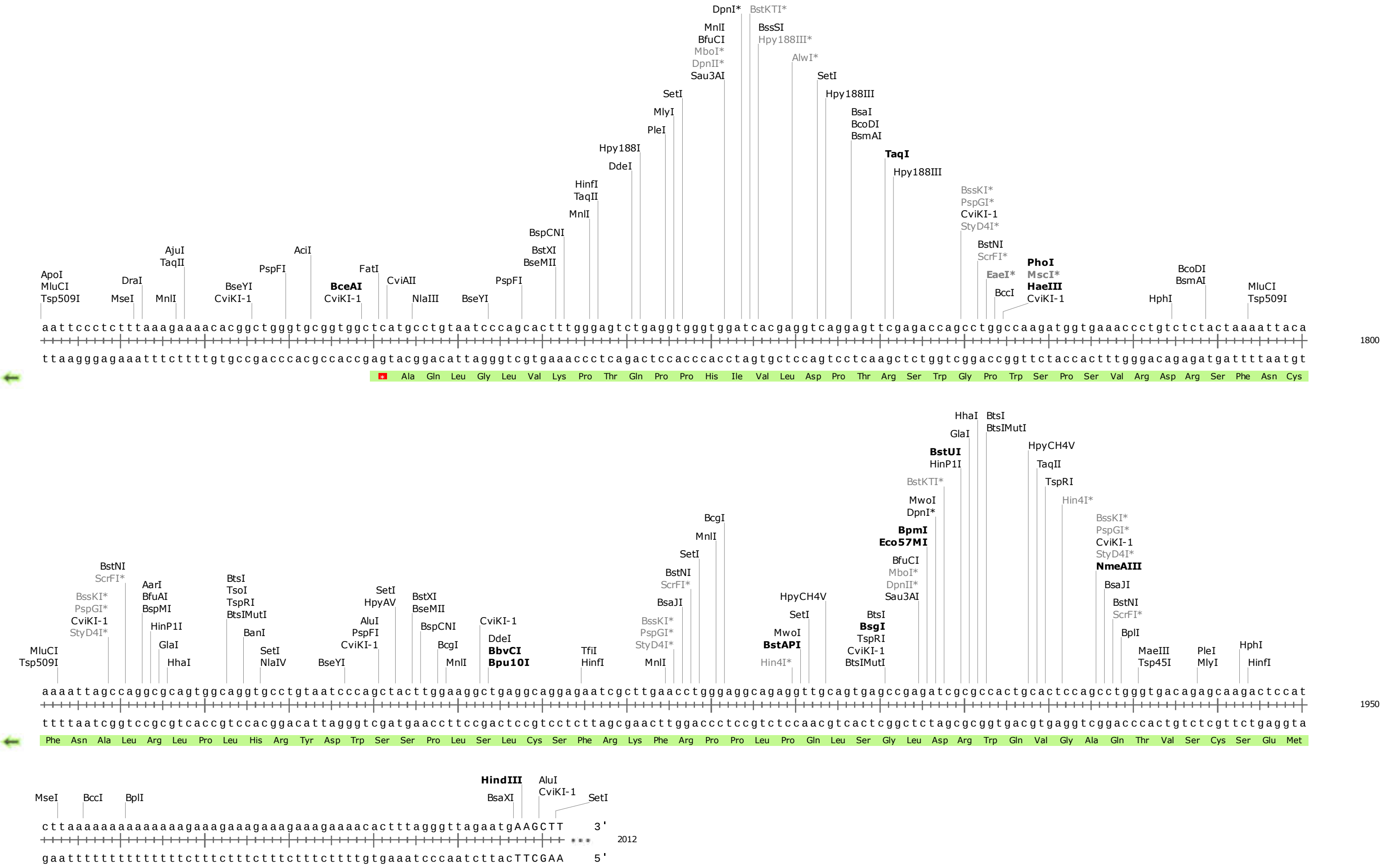

B

QC Items

QC Items	Method	Specifications	Results
Target Sequence	Sequence alignment	Sequencing results are consistent to the confirmed sequence.	Pass
Vector Sequence	Sequence alignment	20bp flanking sequences of the vector are correct.	NA
Reading Frame	Sequence alignment	Frame is correct and consistent to the client's requirement.	Pass
Fragment Size	Restriction Digests	The size of inserted fragment is right and free of any contaminated bands.	Pass
Quality/Quantity	UV spectrophotometry	Miniprep: OD260/280=1.7~2.0<br>4μg/tube, 1 tube, Lyophilized	Pass
Appearance	Visual inspection	Clear and free of foreign particles.	Pass
Customized Test	NA	NA	NA

Restriction Digestion Map

M 1 2

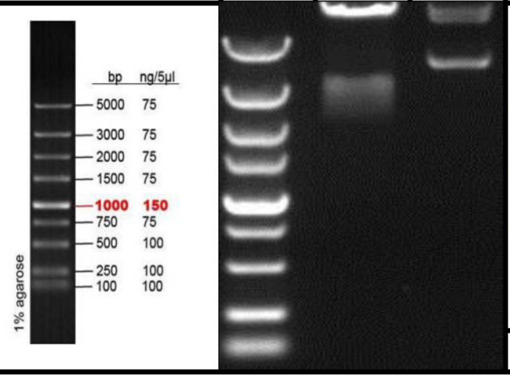

Lane M: DNA Marker

Lane 1: Plasmid digested by KpnI HindIII

Lane 2: Plasmid DNA

Date: 2021/7/25

Description:
