# Supplementary material for: Function of PHEX mutations p.Glu145* and p.Trp749Arg in families with X-linked hypophosphatemic rickets by the negative regulation mechanism on FGF23 promoter transcription
Source: Cell Death Dis. 2022 Jun 2;13(6):518. doi: 10.1038/s41419-022-04969-5 (PMC9163062; doi:10.1038/s41419-022-04969-5)
Supplement: Supplementary file 6 — Supplementary file legends [file 41419_2022_4969_MOESM6_ESM.docx]

**Supplement 1.** The cloning sequence of pBoBi-N-3*Flag *PHEX*.

**Supplement 2.** the cloning sequence of pBoBi-N-3*Flag-sec*PHEX*.

**Supplement 3.** a, The cloning sequence of pGL3-Basic-*hFGF23* promotor. b, Dual luciferase reporter vector plasmid identified by double enzyme digestion. Lane 1 is plasmid digested with KpnI-HindIIILane 2 is plasmid DNA and Lane M is DNA marker.
